# Disclosure of symptoms of postnatal depression, the perspectives of health professionals and women: a qualitative study

**DOI:** 10.1186/1471-2296-10-7

**Published:** 2009-01-21

**Authors:** Carolyn A Chew-Graham, Deborah Sharp, Elizabeth Chamberlain, Liz Folkes, Katrina M Turner

**Affiliations:** 1 School of Community-Based Medicine, University of Manchester, and National School for Primary Care Research, National Institute of Health Research, Manchester, UK; 2 Academic Unit of Primary Health Care, University of Bristol; and National School for Primary Care Research, National Institute of Health Research, Bristol, UK; 3 Centre for Women's Studies, University of Manchester, Manchester, UK

## Abstract

**Background:**

In the UK, 8–15% of women suffer from postnatal depression with long term consequences for maternal mood and child development. Current guidelines state that health visitors and GPs should continue to have a major role in the detection and management of postnatal depression. Previous literature suggests that women are reluctant to disclose symptoms of postnatal depression. This study aimed to explore general practitioners' (GPs), health visitors' and women's views on the disclosure of symptoms which may indicate postnatal depression in primary care.

**Methods:**

In-depth interviews with GPs, health visitors and women who were participating in a randomised controlled trial of anti-depressants versus health visitor delivered non-directive counselling for the treatment of postnatal depression. Interviews were audio-taped and fully transcribed. Thematic analysis with an iterative approach was used, allowing the views of practitioners and patients to be explored and then compared.

**Results:**

Nineteen GPs, 14 health visitors and 28 women were interviewed. A number of common themes were identified across all three data sets: understanding and negotiating the diagnosis of postnatal depression, hindering and facilitating disclosure, and the system of care. Both women and health professionals described postnatal depression in psychosocial terms: an adjustment reaction to change in life circumstances and the reality of motherhood not meeting personal expectations. Women described making a conscious decision about whether or not to disclose their feelings to their GP or health visitor. Health professionals described strategies used to hinder disclosure and described a reluctance to make a diagnosis of postnatal depression, as they had few personal resources to manage women with postnatal depression themselves, and no services to which to refer women for further treatment.

**Conclusion:**

To improve disclosure of symptoms in primary care, there should be a move away from questioning why health professionals do not make the diagnosis of depression and in response suggesting that education and training will improve skills and thus improve detection of depression. Improving the detection and management of postnatal depression in primary care requires recognition of the context in which women consult, and system changes that ensure health professionals work in an environment that can facilitate disclosure and that the necessary resources for management are available.

**Trail Registration:**

ISRCTN 16479417

## Background

Postnatal depression is one of the core diagnoses in the National Service Framework (NSF) for Mental Health[1] in the UK. Postnatal depression is a non-psychotic depressive episode meeting standardised diagnostic criteria for a minor or major depressive disorder, beginning in or extending to the postnatal period[2]. It represents a substantial public health problem affecting 8–15% of women and can result in long-term adverse consequences for maternal mood and infant development[3]. An episode of major depression may have detrimental consequences for the family, erode already difficult marital relationships and reduce confidence in parenting. Even mild to moderate postnatal depression can have such effects[3] and, in addition, postnatal depression leads to increased health service resource utilisation[4].

Most women with postnatal depression are treated in primary care, with only very severe cases or women with psychosis or suicidal intent being referred to secondary care. The recently published NICE guidelines[5] for antenatal and postnatal mental health use the term "perinatal mental disorder", recognising that symptoms may begin in the antenatal period, and outline the potential role of healthcare professionals in the detection of depression. The guidelines suggest the establishment of specialist multidisciplinary perinatal services in each locality but that, until the commissioning of such services, health visitors and GPs should continue to have a major role in the detection and management of perinatal depression.

Previous qualitative studies have depicted the feelings and experiences of mothers with postnatal depression[6]: "loneliness, obsessive thinking, anxiety attacks, loss of control, guilt, insecurities, diminished concentration, fear that life would never be normal again, lack of positive emotions, loss of interests in hobbies or goals, and fear of contemplation of harming themselves and their infants" (Beck, 1993 p. 43). From an empirical perspective, mothers' descriptions of postnatal depression also include an element of loss, such as loss of control or loss of former identity[7]. Oates[8] suggests that childbirth is a time of psychological and social change and alerts clinicians to the difficulties of making the diagnosis of postnatal depression both clinically and attempting to integrate the use of the rating scales, such as the Edinburgh Postnatal Depression Scale (EPDS) into routine clinical practice. In a qualitative systematic review, Dennis and Chung-Lee[9] described women's experiences of help-seeking and identified that a common barrier to accessing help was women's inability to disclose their feelings, which was reinforced by family members' and health professionals' reluctance to respond to the mothers' emotional and practical needs. Although Dennis and Chung-Lee discussed significant health service barriers, factors in the consultation were not implicated.

For over 40 years, GPs have been accused of failing to diagnose depression[10,11]. Oates suggests the view of a substantial morbidity due to undiagnosed depression in primary care needs to be challenged^8 ^and other studies suggest[12] that clinically significant depression (moderate to severe depressive illness) is detected and it is milder forms that go undetected and un-treated, and these remit spontaneously. It is argued[8] that the importance of adequately diagnosing and treating clinically significant postnatal depression is centred upon two issues. First, the context distinguishes depression at this time from depression at other times. There is an expectation of happiness and fulfilment. Depression following childbirth is particularly distressing, interferes with the adjustment to motherhood, leaves lifelong memories of unhappiness and guilt and can profoundly affect relationships, particularly marriages and other children[8]. Second, postnatal depression, particularly if untreated and chronic, combined with social adversity and marital conflict, has measurable adverse effects both in the short and long term on infant development, particularly for boys[8].

Whilst there is a broad literature on women's disclosure of domestic violence to health professionals[13,14], particularly health visitors, few researchers have considered the more general issue of disclosure of depressive symptoms postnatally to clinicians. Zink[15] reports that questions about sexual risk and depression create the most discomfort for both women and family physicians. Furthermore, depression can be shrouded with the stigma of mental illness[16] and women may prefer to talk to friends rather than professionals about their feelings postnatally[17].

There is no previous literature comparing the contemporaneous views and attitudes of women and health professionals about the disclosure of postnatal depression in primary care. We conducted in-depth interviews with GPs, health visitors and women who had been diagnosed with postnatal depression. Respondents described their views about how disclosure of symptoms of postnatal depression might be facilitated or hindered during the primary care consultation.

## Methods

This study was approved by Scotland A MREC Committee (MREC/03/0127), three local research ethics committees and research governance agreement from participating Primary Care Trusts (PCTs) in Bristol, Manchester and London.

Interviews were undertaken as part of a multi-centre pragmatic randomised controlled trial [the RESPOND trial **]. Nine PCTs participated in the trial in Bristol, Manchester and London, and general practices were invited to opt-into the study. The nine Trusts covered inner city and urban areas, with some areas of very high levels of deprivation, particularly in Manchester. Sure Start programmes[18] were located in pockets of the participating Primary Care Trusts in Manchester and London. For the trial, practice administration staff sent out packs containing the Edinburgh Postnatal Depression Scale (EPDS) to women who had delivered four weeks previously. Women who returned the screening EPDS and scored over 11 were contacted and visited by the researcher who confirmed (or excluded) the diagnosis of postnatal depression using the CIS-R [Clinical Interview Schedule (Revised)].

In-depth interviews were carried out with a purposive sample of GPs and health visitors who were involved in the RESPOND trial. Their purpose was to explore the attitudes GPs and health visitors held towards women with postnatal depression, its diagnosis and management in primary care. The sample was drawn from GPs and health visitors participating in the trial. Sampling was purposive and sought to achieve maximum variation in relation to GPs' age, gender, length of time in general practice, practice size and level of deprivation. Sampling of health visitors was purposive so that variation was achieved in relation to time since completion of training and length of service.

The in-depth interviews with health professionals were conducted between January 2006 and February 2007. Interviews were carried out by EC and LF (non-clinical research associates on the study). Nineteen GPs and fourteen health visitors were interviewed at their place of work, and interviews lasted between 25 and 67 minutes. An interview guide provided a flexible framework for questioning and explored a number of topics: models of postnatal depression; diagnosis and management of postnatal depression; the patient-professional relationship; professional-professional relationships and ways of working. The interview guide included open questions to elicit free responses, and more focused questions for probing and prompting. Interviews were audio-recorded and transcribed verbatim.

Women who had taken part in RESPOND and completed their final outcome measures at 44 week post-randomisation were invited to participate in the qualitative study. One woman who had consented to the initial assessment for the trial but then declined randomisation also agreed to be interviewed. When sampling women who had completed the trial, a purposeful sampling approach was used to ensure interviews were held with women randomised to different treatment arms and living in different cities. Twenty eight women were interviewed between November 2006 and June 2007. All of them were interviewed by LF. Participants were asked to describe their views on the causes of postnatal depression, and their experiences of treatment. A flexible interview guide was used to ensure consistency across the interviews, whilst allowing respondents to raise issues salient to them.

Twenty eight women were interviewed between November 2006 and June 2007. Two respondents were interviewed over the telephone. The remaining women were interviewed face-to-face in their own homes. The interviews lasted between 40 minutes and 2 hours. All were audio-recorded and fully transcribed.

Analysis proceeded in parallel with the interviews, allowing for modification of the interview guides in the light of emerging themes. Analysis was inductive and a thematic approach taken[19]. Transcripts were read and discussed by researchers from different professional backgrounds[20] (primary care, psychology, nursing). Coding was informed by the accumulating data and continuing thematic analysis. Thematic categories were identified in initial interviews, which were then tested or explored in subsequent interviews where disconfirmatory evidence was sought[19]. Interpretation and coding of data was undertaken independently by all authors and with themes agreed through discussion. Analysis across the data sets allowed comparisons to be made between the views of the health professionals with those of the women.

In reporting the final analysis, data were presented to illustrate the range and commonality of meaning of each category of analysis from the perspectives of GPs, health visitors and women. In presenting the data, similarities and differences between professionals' and women's accounts were noted.

## Results

A number of themes emerged from the analysis: understanding postnatal depression, making the diagnosis, facilitating and hindering disclosure and the role of the system in inhibiting disclosure of symptoms. In this paper, when reproducing quotes, a unique identifier has been given in order to indicate the study location, respondent's profession (for GP or health visitor) or unique ID number (for the women).

### Understanding postnatal depression

Women attributed a psychosocial aetiology to their symptoms in relation to the stresses of parenthood, such as changed relationships, reality not meeting expectations, and the birth of the child triggering memories of past events:

*"And then there was a whole set of issues about my relationship with my partner and how he was supporting me and these issues were all thrown up when my little girl was born and then when my little boy was born, it happened exactly the same. It was a real repeat, you know, and I'd done so much to try and prevent it going down the same route, but I just felt like we were going down the same groove." *(B ID28)

Some women described their feelings as a response to physical changes around childbirth, but suggested that they were susceptible to depression in some way:

*"I personally feel it ... well, probably two factors...but I think perhaps it is some sort of chemical hormone or imbalance, you know, everything sort of all shifts it about and ... and secondly, just the type of character that I am, and putting that pressure on myself the whole time." *(L ID16)

Women described insights into and awareness of their symptoms, often because they had suffered from depression in the past, although they suggested that the cause of postnatal depression might be different to the cause of previous episodes:

"*I'd had slight depression before, but this was quite different, I kind of knew why, whereas before it was kind of all suffering and something had triggered lots of other things, and here it was lack of sleep, and although I knew the reasons why I was feeling like I was feeling, but I couldn't stop it." *(B ID3)

Health professionals also attributed a psychosocial aetiology to postnatal depression and demonstrated ambivalence about the status of postnatal depression as a separate condition as compared with depressive illness at other times in a woman's life:

"*I call it emotional turmoil rather than depression....psychological disturbance, at various stages after the birth, and I don't think of them as adjustment disorders, and often they are what I would think of as existential crises*." (M GP1)

"*I can certainly give you a list of things that would put women at risk, but, you know, clearly doesn't always result in postnatal depression. So a previous history of mental health problems or depression, unfulfilled expectation, difficult birth, wrong sex, partner unsupportive...but, I think there's quite a large proportion where there appears to be no risk factors*." (B HV2)

Thus, both women and health professionals viewed the cause of postnatal depression as multifactorial and often a social response to birth.

### Making the diagnosis

GPs and health visitors described a reliance on instinct or clinical intuition, which would alert them to the possibility of postnatal depression, rather than using formal screening instruments or actively seeking out symptoms of depression:

"*So I'm not saying I actively look for it, but I am hoping my antennae would tell me if there was a problem*." (M GP5)

"*I think any kind of flatness...it's a difficult thing to explain, isn't it?...You can just tell by having a conversation...just chatting to them*." (B HV1)

Most health visitors described a reluctance to make a diagnosis of postnatal depression:

"*I'm reluctant to say postnatal depression because I'm not in a position to actually diagnose PND*." (M HV2)

This may indicate that the health visitors felt their own professional position was subordinate to that of GPs, not being diagnosticians, rather than having an equal role in the division of work in primary care:

*"...it's not actually post-natal depression unless it's been diagnosed by the GP. That is 'cos, we have total care, which is, you know, a computer input system and we only input people where there are concerns, and one of them might be post-natal depression, but you only input them as being postnatally depressed if it's been diagnosed." *(L HV5)

GPs described difficulties in using the label for postnatal depression with women, particularly referring to the stigma that they perceived women felt, and the effect on this on the consultation:

*"I mean, if they deny that they have got a problem but are still in tears, it becomes very difficult, because you can't treat somebody if they don't accept that there's something to treat." *(B GP1)

Other GPs, however, described consultations where the woman was happy to accept the label:

*"...and equally others will just come in and say 'my husband said I've got to get this sorted out, and I need a tablet to calm me down' or whatever. You get the whole spectrum, really." *(M GP2)

Health visitors did not describe such dilemmas within their consultations with women, although recognised the reluctance women might feel to accept the label of depression and the anticipated treatment:

*"But yes, I've found there are many clients who don't want to take medication, because they do think there is a stigma attached." *(L HV5)

Some GPs described a reluctance to use the term postnatal depression because they felt that symptoms would recover without formal interventions, because of a lack of services or referral options, and the feeling that antidepressants were the only treatment option available:

*"I don't want to medicalise it too much really. I think it needs to be a sort of informal sort of network because I do think most of the time people do recover from it if they are just given some support rather than medication." *(M GP8)

"*If I call it depression, I need to do something. There's no one to refer to, so I would rather call it something else and manage her myself." *(M GP10)

*"I mean, it's best if it's a multiple approach rather than just drugs. Unfortunately that's all we can offer." *(L GP1)

Health professionals described a variety of difficulties making the diagnosis of postnatal depression; health visitors because diagnosis was not felt to be within their remit, and GPs because of the perception that they had limited management options to use if they used the label of postnatal depression.

### Disclosure and hindering disclosure

Women described making a conscious decision about whether or not to disclose their feelings to their GP or health visitor. Some women cited their own personal barriers to being able to talk to their GP, whilst others mentioned characteristics of GPs which inhibited their ability to disclose, such as the GP being perceived as not willing to listen:

*"...he *[GP] *could have listened. Again, I think they could have done that at least, they could have listened..." *(M ID22)

Other women described system factors which made them reluctant to attempt to approach their GP to discuss how they were feeling:

"... *wouldn't go to the doctors because you can never get an appointment and it's crap. They always treat you like there's something else wrong and why are you wasting his time....I wouldn't have gone *[to the doctors] even if *I'd been dragged kicking and screaming*." (M ID24)

Many women described a fear of disclosure because of how they would be perceived by their health visitors, such as being a bad mother, and others described fear of having their children being referred to social services:

*"...with my health visitor, I, I try not to, try not to let too much out because then she won't think I am a bad mum, if you see what I mean, so I tend not to let too much out with the health visitor." *(B ID2)

Women also questioned the role of the health visitor and who she was there for:

"...*what ****is ****the health visitor there for? Is she there for the welfare of the child, or is she there for the welfare of the mother, or both?" *(M ID24)

Other women described a fear of consulting their GP as they anticipated that the only treatment that would be offered would be antidepressants, which they did not feel were an acceptable treatment option:

*"That's all they have, GPs, and I just didn't want to go onto antidepressants, because obviously I've heard people get addicted to them and then you're stuck on them and you have a vicious circle." *(M ID24)

*"My concern is that I will just get addicted and it will change my personality." *(B ID1)

This view might be reinforced by the women's health visitors, as the health visitors were interviewed described GPs' role in the management of postnatal depression as being limited to the prescribing of antidepressants:

"*The GPs.....and I think they're even worse than we are because they....from experience of women I've visited, they just write a prescription, put them on anti-depressants..." *(HV M2)

*"...sometimes the GPs are, they don't have a very sympathetic attitude to postnatal depression, let's say. And so I would imagine it puts the mothers off going to see them actually." *(HV L5)

A few women did talk about why they felt able to discuss their symptoms with the GP and cited factors such as having been to their GP with depression or postnatal depression in the past and recognising the symptoms. Women who had previous experience of antidepressants were more accepting of this treatment being offered to them again:

"*I thought, well, I'll try them and you know, it did help a bit last time, not, you know, it wasn't fantastic, but it did help a bit so I thought well, okay, I'll try them again." *(B ID6)

Most importantly, those women who did feel comfortable seeking help from their GP described having a good relationship with him/her, making discussion of depression possible:

"*I don't go to the doctor that often but, well *[names child] *was with *[names doctor]*so I've been quite a few times with having colds and stuff so I knew him quite well, so it was quite easy to go and say 'look, I'm just, I'm not feeling right at the moment'." *(M ID25)

Some GPs described strategies used to facilitate disclosure and offer women ongoing support:

*"Once you kind of know they're in distress you don't just give them one session, you ask them to come back always...you get them to come back two weeks later to see how they're doing." *(L GP1)

In addition, a few of the health visitors described exploring depressive symptoms as a routine part of their interactions with women:

"...*but I think most people have actually heard about it now and don't find it unusual that you ask them about their mood and how they are feeling." *(B HV2)

Most health visitors, however, suggested that there was limited value in identifying women with postnatal depression as all they could do was to refer a woman to the GP and they viewed the GP's role as limited to the prescribing of antidepressants:

"*So, there's almost an ethical dilemma of, well, is there any point in identifying them if you can't do anything with them other than send them to the GP for anti-depressants, which isn't good, you know*." (M HV5)

This view pervaded the health visitor transcripts and was reported by the health visitors to be a major reason why health visitors did not encourage women to disclose their feelings to their GP.

### How the system of care hinders disclosure

Both GPs and health visitors, however, described the current systems of care as hindering disclosure of symptoms of postnatal depression:

*"You know, we're not user-friendly in the health services...say someone is de-motivated because of low mood, then they ring for an appointment and they can't get through and then we ask patients 'do you really need to be seen today?'...they have to jump through hurdles." *(B GP6)

*"They know it's a hurried environment, and they know it's hardly the environment for them to give you clues or confide in you that they're depressed." *(M HV5)

Some health professionals described consciously inhibiting disclosure in order not to be placed in this position citing lack of continuity of care as the reason:

*"Easier not to ask, if I'm not going to see her again." *(L GP1)

*"For the vast majority of them we don't (see them again), we're not able to offer even a routine follow up visit, even if it's their first baby. We explain the situation to them in terms of staff and resources, and we encourage them to come to clinic or to phone us, and we explain that we do visit some families at home and offer them extra support...however, in terms of listing the priorities I would say the post-natal depressed ones aren't high up on the agenda. When we've got much more prioritised." *(M HV5)

*"... but I think they used to get to know us, and we used to get to know them and obviously if they know someone they're more likely to sort of be forthcoming with any problems aren't they? Whereas now they, they probably don't get that input so they're probably less likely to come forward with things*." (M HV4)

A few of the health visitors described a resistance to being involved in providing care for women with postnatal depression, and this reluctance was made easier by the move to corporate working:

"*Working corporately, we all work between the GPs and as work comes in we allocate families...... I mean the families aren't becoming reliant on you, that's the good thing*." (M HV1)

"*You cannot emotionally and mentally prop somebody up for years and years...sounds awful, doesn't it?" *(B HV4)

Some women suggested that neither their GP nor their health visitor could do very much, which resonates with health visitors' views on the GP role:

"*There is nothing else available. No GP, no health visitor; they're there but not in a helpful sense, sort of like." *(M ID22)

*"...because I was very struck by the health visiting that I'd had so far. I hadn't felt like my needs had been met at all." *(M ID27)

*"The doctors are like, always helpful, but I think they're limited as to what they can do, which is what I thought, you know...that they can't actually do that much..." *(B ID6)

Both GPs and health visitors described organisational factors which precluded the facilitation of the disclosure of depressive symptoms in their postnatal women patients. Difficulties providing continuity of care were frequently cited. Women, talked less about system factors, but rather suggested that neither the GP nor health visitor had much to offer. The development of long-term relationships which may facilitate disclosure may be impeded by the systems within which health professionals currently work.

## Discussion

### Summary of main findings

Our data suggests that health professionals and women conceptualise postnatal depression in similar ways citing psychosocial factors (including reality not meeting expectation, adjustment to new role, motherhood stirring up things from the past) as the main cause of their symptoms, and feeling that management of postnatal depression needs to address these factors. Our findings suggest that many women decide not to seek help with their symptoms and distress if they predict that medication will be the only treatment offered to them. GPs and health visitors do not use the label "postnatal depression" if they feel they personally have nothing to offer the woman, or no services to refer women on to. Some health visitors view GP management as limited to the prescribing of antidepressants. Health visitors and GPs make conscious decisions in their everyday work about whether or not to facilitate women's disclosure of symptoms of postnatal depression. In addition, health visitors refer to the new way of working – that of working corporately with no personal list of women, no relational continuity and no responsibility for individual women – as hindering the disclosure of symptoms.

### Strengths and Limitations

Data were gathered from GPs, health visitors and women drawn from a large geographical area (9 Primary Care Trusts in 3 major UK cities), which included areas of varying levels of socio-economic deprivation. The use of qualitative methods allowed respondents to raise issues that were of concern to them, and the inductive analysis approach ensured findings were related to the views articulated. Using researchers from different professional and academic backgrounds to analyse the data is a recognised technique for increasing the trustworthiness of the analysis[20].

Only health visitors, GPs and women who were already involved with the RESPOND trial were interviewed [being a stipulation of the Ethics Committee]. The Primary Care Trusts participating in the RESPOND trial did not have a well established pathway of care for postnatal depression, thus participating PCTs may have poorer provision of services and the attitudes of the health professionals working in these areas may reflect this. The findings may, therefore, not be representative of health professionals in (even neighbouring) Primary Care Trusts which may have developed a postnatal depression strategy and services for this group of patients. Generalisability of the findings will also be limited by the fact that both women and practitioners were purposively sampled. In addition, because this nested qualitative study was nested in a trial, women were sampled and interviewed after the final outcome measures were made at 44 weeks, so women's responses in the interviews were subject to recall bias and represent views and constructions looking back and coloured by experience in the RESPOND trial.

### Comparisons with existing literature

Our data confirms previous literature that suggests women conceptualise postnatal depression in the context of role transition, loss of familiarity and loss of control[21]. The construction of 'depression' as a clinical condition is contested amongst GPs[22-24] but there is little published work on the role played by GPs in the management of postnatal depression. Our study suggests that postnatal depression is perceived as a reaction to birth with psychosocial rather than having a biomedical aetiology. Previous evidence that health visitors conceptualise postnatal depression as an understandable result of parenting in a hostile world[25], and our data supports this view. There is a reported confusion on the part of health visitors as to who is the patient[25], and women in our study echoed this confusion, not knowing if the health visitor was for them or their baby.

Maxwell describes the social and moral reasoning that lies behind women's decisions to seek help for symptoms of postnatal depression, and to subsequently accept their GPs' explanation and advice, and that the acceptance of antidepressants created a moral dilemma for the women[26], confirmed in other studies[27]. Patients have difficulty in presenting their distress and discussing their concerns with their doctor, especially when they are uncertain that depression is a legitimate reason for seeing the doctor[28]. The MaGPIe Research Group[29,30] suggests that the relationship is important, and that GPs are, in fact, effective at identifying mental health problems in patients they know and that some people believe that the GP is not the right person to talk to, or that such symptoms should not be discussed at all. Women in our study suggested that depressive symptoms were a legitimate reason for seeking care, but they felt unable to do so because of perceptions about access, the skills of the GP in discussing depression and the limited treatment options they thought would be offered. There is evidence that patients with psychosocial problems and medically unexplained symptoms give cues to the GP about their distress in the primary care consultation[31]. Our data suggests that patients consciously decide whether or not to give these cues.

Whilst disclosure of domestic violence is reported to be linked to the setting of the encounter[14], there is little published work on women's decisions to disclose depressive symptoms to health visitors. Zink[15] describes how women feel more comfortable discussing domestic violence than symptoms of depression, and that a positive ongoing relationship with the health visitor is important to facilitate disclosure of depressive symptoms, something that women and health visitors in our study found hard to develop. Women expressed concern about how they would be viewed by the health professional. They reported fears that they would be viewed as being a "bad mother" and that they would be judged as not fit to care for their child. These fears, together with the confusion about whether the health visitor was there for themselves or their baby, were reported to influence women in consciously making the decision not to disclose their distress to either their health visitor or GP. Thus health visitors and GPs must develop a trusting relationship with patients, know how to communicate and how to listen to and discuss sensitive matters including depression. Whilst there may be training issues that need to be addressed [32], the development of long-term relationships which respondents feel are necessary may be impeded by the systems within which health professionals currently work. The role of the organisation within which these health professionals work was cited as a major barrier to encouraging disclosure of symptoms and a lack of resources, both personal and places to refer women to, added to professionals' reluctance to encourage women to disclose their distress, echoing previous work[33,34].

This study suggests that focussing on the role of the health professional and questioning why a diagnosis of depression is not made, and suggesting training for the health professionals, is too narrow an approach. Instead, we suggest that a whole system approach[35], with intervention at the level of the patient, practice and extended primary care team, will be necessary to improve women's willingness to disclose, health professionals' ability to listen and intervene, and a system that will facilitate management of women with postnatal depression.

## Conclusion

Improving the detection and management of postnatal depression in primary care requires recognition of the context in which women consult, and system changes that ensure health professionals work in an environment that can facilitate disclosure and that the necessary resources for management are available.

## Competing interests

The authors declare that they have no competing interests.

## Authors' contributions

CCG designed and managed the health professional element of the qualitative study. She contributed to the data analysis and drafted the paper. She is guarantor for the study and paper. DS is Principal Investigator on the RESPOND trial and contributed to analysis and writing of the paper. EC contributed to the design of the study and interview schedules, collected and analysed data, and contributed to the writing the paper. LF contributed to data collection and analysis. KT designed and managed the patient element of the qualitative study, analysed data and contributed to the writing of the paper.

## Pre-publication history

The pre-publication history for this paper can be accessed here:


